# Confirmation of “pre-plasmolysis mediated ex-osmosis hypothesis” to obtain shoot bud morphogenesis in *Catharanthus roseus*

**DOI:** 10.1186/s43141-021-00170-x

**Published:** 2021-05-07

**Authors:** Vyoma Mistry, Abhishek Sharma, Ajay Kumar Mathur

**Affiliations:** 1 C. G. Bhakta Institute of Biotechnology, Uka Tarsadia University, Gopal-Vidyanagar, Maliba Campus, Surat, 394350 India; 2 Department of Plant Biotechnology, Central Institute of Medicinal and Aromatic Plants (CIMAP), Council of Scientific and Industrial Research, PO CIMAP, Lucknow, 226015 India

## Abstract

The antineoplastic herb, *Catharanthus roseus* is a classified high-value low-volume medicinal herb which is in global attention of scientific research for modulation of its monoterpenoid indole alkaloids (MIA) pathway through genetic engineering. These secondary metabolites are generally stored in specific types of structures/compartments due to their cytotoxic nature and designated roles in plant defense response. However, their presence can hinder the genetic engineering process used to develop transgenic plants through de novo morphogenesis and regeneration of plants from cultured cells/tissues and hence, it always remained a critical impediment in transgenic research in *C. roseus*. The pre-plasmolysis treatment of leaf explants can help to tackle the recalcitrant nature of leaf explant and can support the direct regeneration response by ex-osmosis that minimizes the concentration of alkaloids. Therefore, this study was performed to chase the effect of osmotic conditions on recalcitrant leaves of *C. roseus* engaged in vitro plant regeneration and hypothesis of alkaloids ex-osmosis is confirmed by HPLC analysis.

## Introduction

*Catharanthus roseus* is an important medicinal plant due to its property to synthesize two anticancer phytomolecules monoterpenoid indole alkaloids (MIAs) vinblastine and vincristine [10, 11]. In the Indian traditional medicinal system, *C. roseus* plant parts like seeds, leaves, flowers, and roots are often used to cure diabetes, hypertension, menorrhagia, and tumor growth, whereas in the modern pharmacopeia, the plant is appreciated for two leaf-derived antineoplastic dimeric MIAs vincristine and vinblastine and root-derived antihypertensive monomeric MIAs ajmalicine and serpentine [3, 12]. However, the *in planta* production and availability of antineoplastic vinblastine and vincristine are highly inadequate. Also, the absence of alternative chemical synthesis has enhanced their pharmaceutical industry demand and made exorbitant market cost [9].

Therefore, several plant tissue culture systems viz. suspension and hairy root cultures, elicitation, and especially genetic engineering studies were carried out on *C. roseus* to enhance the production of these pharmaceutically important molecules. However, suspension and hairy root culture systems failed to enhance antineoplastic MIAs due to their limited cellular-organ specific complexity, whereas *C. roseus* due to its recalcitrant nature was not found a very acquiescent system for whole plant genetic engineering through plant regeneration-based transgenic research [11]. Secondary metabolites are generally synthesized for plant defense response and hence may have cytotoxic nature, therefore, stored in the specified cellular sites/compartments. As major, the whole plant genetic engineering attempts were made using leaves, and being the production house of MIAs, leaves always have higher accumulated alkaloid content and may contain some unspecified compounds. These compounds probably impede the cellular dedifferentiation and redifferentiation and can be the reason for the recalcitrant nature of *C. roseus* leaves. Therefore, de novo morphogenesis and regeneration of plants from cultured cells/tissues has always remained a critical impediment in transgenic research in *Catharanthus roseus*.

Treating the leaves with pre-plasmolysis can help to confront their recalcitrant nature but can also support the direct regeneration response by ex-osmosis of stored alkaloids and toxic molecules from leaves. This applied osmotic pressure will curtail the concentration of toxic phytomolecules inside the growing leaves and/or also can influence the morphogenesis and organogenesis by quick uptake of plant growth promoters from culture media through an osmotic pull [14]. Therefore, the present study is performed to chase the effect of pre-plasmolysis treatment on leaf explants before culturing over the different combinations of culture media to obtain a direct shoot bud organogenesis protocol. The obtained results will help to confirm and correlate the ex-osmosis hypothesis through HPLC based detection of MIAs and leaf surface histological studies.

## Methods

### Leaf explant source

Whole leaves (2.0–3.0 × 1.0–1.5 cm) from glass-house grew plants or 6–8-week-old in vitro grown multiple shoot cultures of the two *C. roseus* genotypes (“Dhawal” and “Nirmal” having National Gene Bank Accession Numbers (CIMAP-0859), and (CIMAP-0865), respectively) were used as explant in these experiments.

### Pre-plasmolysis treatment and organogenesis

The leaf explants, before their culture over regeneration medium, were pre-plasmolyzed for 15, 30, 60, and 90 min in a high osmotic (hypertonic) solution prepared by adding different concentration (5–20% w/v) of mannitol in the cell protoplast washing (CPW) [4] solution (Table 1). The pre-plasmolyzed leaves were given a quick wash in sterilized distilled water followed by in basal liquid MS [8] medium, blot dried, and placed horizontally with their adaxial surface in contact with the half- or full-strength woody plant medium (WPM [6];) with several combinations of BAP (1.0 to 7.5 mg/l) and NAA (1.0 to 5.5 mg/l). Shoot bud regeneration frequency and the rate was expressed as the mean percentage of explant responded and the number of shoot buds induced per responsive explant, respectively.

**Table 1:** Composition of cell protoplast washing (CPW) medium

S. No.	Component	Concentration (mg/l)
1.	KH_2_PO_4_	27.2
2.	KNO_3_	100
3.	CaCl_2_·2H_2_O	150
4.	MgSO_4_·7H_2_O	250
5.	KI	0.16
6.	Cu SO_4_·5H_2_O	0.025

### Alkaloids extraction and HPLC quantification

HPLC analysis of the hypertonic solutions used for pre-plasmolytic treatment was carried out to trace the presence of MIAs in this pre-plasmolytic solution and to support our hypothesis of ex-osmosis of alkaloids during pre-plasmolytic treatment. For HPLC quantification, the hypertonic solutions used for pre-plasmolytic treatment were air-dried and remnants were dissolved in methanol and analyzed through high-performance liquid chromatography (HPLC) for the determination of monomeric alkaloids vindoline and catharanthine and dimeric alkaloids vinblastine and vincristine. Shimadzu Prominence-I, LC-2030 plus gradient and auto-inject HPLC system with Shimadzu RP-18e reverse-phase HPLC column was used for HPLC analysis. For mobile phase, acetonitrile to ammonium acetate (100 mM, pH 7.3) (50:50) was used and detection was done at 254 nm.

### Microscopic examination of leaf epidermis

Epidermis of control and pre-plasmolyzed leaves were peeled off using fine forceps and stained with safranine (1% w/v) before mounting in Canada balsam. Stained sections were viewed and photographed on a high-resolution microscope (Lyka S8-APO).

### Experiment design and statistical analysis

Pre-plasmolytic experiments were performed in a completely randomized design (CRD), performed three times with a minimum of 50 healthy leaves for each treatment. All data represented as a mean ± standard deviation (SD). Statistical differences between results for pre-plasmolytic experiments were evaluated by two-way analysis of variance (ANOVA) and post hoc Tukey’s HSD test using SPSS V. 17.0, and the values at *p* < 0.05 were considered statistically significant.

## Results and discussion

### Leaf pre-plasmolysis and direct shoot bud organogenesis

Out of different concentrations of BAP (1.0 to 7.5 mg/l) and NAA (1.0 to 5.5 mg/l) used with half- or full-strength woody plant medium (WPM), the full-strength WPM with 4.5 mg/l BAP + 2.5 mg/l NAA was found most suitable to provide direct shoot bud formation, however, with a delayed response of 60–65 days. To further improve the shoot bud regeneration response obtained on direct regeneration medium, the effect of a pre-plasmolytic treatment on leaf explant before subjecting them to a direct regeneration experiment was evaluated in the present study. *The rationale behind this approach was to facilitate the possible leaching or dilution of some of the cytotoxic and/or antimitotic metabolites from the explant tissue that might be hindering the in vitro de novo morphogenesis process per se.* To confirm this approach, the leaves of *C. roseus* Dhawal and Nirmal genotypes were subjected to pre-plasmolysis treatment for 15, 30, 60, or 90 min in 1, 10, 13, 15, and 20% (w/v) mannitol-fortified CPW solution before culturing them onto direct regeneration media (Table 2). In pre-plasmolytic treatment of 30 min in CPW, 13% mannitol solution before culturing on shoot bud induction media provided effective results with the development of 1.46 ± 0.09 and 1.31 ± 0.11 well-organized shoot buds from leaves of Dhawal and Nirmal genotype, respectively. More than 80% of explants resulted with direct shoot bud regeneration directly from leaf without any intermediate callus phase. In the pre-plasmolysis treatment with CPW, 13% mannitol also significantly shortened the shoot bud organogenesis period from 50–60 to 30–35 days compared to non-plasmolyzed control leaves.

**Table 2:** Effect of pre-plasmolysis treatment on direct shoot bud regeneration response from leaf explants cultured on shoot regeneration media

Pre-plasmolysis treatment (CPW: mannitol)	Duration of pre-plasmolysis (min)	% of explants showing direct shoot bud organogenesis mean ± SD		Average no. of shoot buds/explants ± SD		Time required for shoot bud appearance on leaf surface (days)	
		cv. Dhawal	cv. Nirmal	cv. Dhawal	cv. Nirmal	cv. Dhawal	cv. Nirmal
**0%**	**0**	**14 ± 1.2**	**12 ± 1.2**	**1.11 ± 0.01**	**1.14 ± 0.05**	**60–65**	**50–60**
**1%**	**15**	**40 ± 3.6**	**17 ± 1.6**	**1.16 ± 0.03**	**1.07 ± 0.04**	**55–60**	**55–60**
	**30**	**66 ± 7.2**	**27 ± 2.4**	**1.05 ± 0.05**	**1.09 ± 0.05**	**55–60**	**55–60**
	**60**	**70 ± 7.4**	**37 ± 3.4**	**1.13 ± 0.07**	**1.09 ± 0.05**	**45–50**	**45–50**
	**90**	**-**	**-**	**-**	**-**	**-**	**-**
**10%**	**15**	**33 ± 3.6**	**27 ± 2.4**	**1.21 ± 0.07**	**1.13 ± 0.07**	**45–50**	**45–50**
	**30**	**43 ± 4.4**	**37 ± 3.2**	**1.15 ± 0.05**	**1.09 ± 0.07**	**40–45**	**40–45**
	**60**	**-**	**-**	**-**	**-**	**-**	**-**
	**90**	**-**	**-**	**-**	**-**	**-**	**-**
**13%**	**15**	**67 ± 7.2**	**27 ± 2.4**	**1.63 ± 0.12**	**1.28 ± 0.09**	**35–40**	**35–40**
	**30**	**83 ± 9.1**	**43 ± 4.6**	**1.46 ± 0.09**	**1.31 ± 0.11**	**35–40**	**35–40**
	**60**	**-**	**-**	**-**	**-**	**-**	**-**
	**90**	**-**	**-**	**-**	**-**	**-**	**-**
**15%**	**15**	**60 ± 6.8**	**23 ± 2.2**	**1.16 ± 0.07**	**1.43 ± 0.13**	**30–35**	**30–35**
	**30**	**77 ± 8.2**	**40 ± 4.2**	**1.26 ± 0.09**	**1.33 ± 0.11**	**30–35**	**30–35**
	**60**	**-**	**-**	**-**	**-**	**-**	**-**
	**90**	**-**	**-**	**-**	**-**	**-**	**-**
**20%**	**15**			**1.2 ± 0.05**	**1.0 ± 0.03**	**30–35**	**30–35**
	**30**	**-**	**-**	**-**	**-**	**-**	**-**
	**60**	**-**	**-**	**-**	**-**	**-**	**-**
	90	-	-	-	-	-	-

Data expressed as the mean ± standard deviation (SD) with *P* < 0.005

For treatments with CPW, 15% mannitol though was also effective but the regeneration frequency was lesser than in CPW, 13% mannitol treatment. When mannitol concentration was increased to 20%, the regeneration response was drastically inhibited. Treatments longer than that of 15-min duration in 20% mannitol solution proved lethal to explant survival. Control leaf explants treated only with CPW solution (without mannitol) showed poor effect over regeneration response (12–14%). The obtained results of leaves pre-plasmolysis treatment with high mannitol concentration very badly affected the viability and organogenetic responses of leaves [11, 13].

HPLC analysis of the hypertonic solutions used for pre-plasmolytic treatment was carried out to *check the presence of alkaloids in this hypertonic solution and to support our hypothesis of ex-osmosis of alkaloids during pre-plasmolytic treatment*. HPLC analysis reviled the lower but detectable presence of monomeric alkaloids vindoline and catharanthine in hypertonic solution (13%, 15%, and 20% mannitol); however, dimeric vinblastine and vincristine were not detected (Table 3). The highest monomeric alkaloids content was observed in hypertonic solution with 20% mannitol followed by 15% and 13%. Such presence of alkaloids contents in the hypertonic solutions supports the “ex-osmosis hypothesis” of alkaloids from leaves, which minimizes the concentration of alkaloids in leaves thus ensuring a less toxic environment inside the growing cells of leaf explants and undergo cellular dedifferentiation and redifferentiation to facilitate direct shoot-bud organogenesis.

**Table 3:** Alkaloids content in hypertonic pre-plasmolytic treatment solution after pre-plasmolysis of leaves

Pre-plasmolysis treatment (CPW: mannitol)	Duration of pre-plasmolysis (min)	cv. Dhawal		cv. Nirmal	
		Vindoline (mg/kg wt ± SD)	Catharanthine (mg/kg wt ± SD)	Vindoline (mg/kg wt ± SD)	Catharanthine (mg/kg wt ± SD)
13%	15	ND	ND	ND	ND
	30	0.014 ± 0.0001	0.013 ± 0.0001	0.013 ± 0.0001	0.013 ± 0.0001
	60	0.024 ± 0.0002	0.023 ± 0.0003	0.013 ± 0.0002	0.013 ± 0.0002
	90	0.042 ± 0.0004	0.051 ± 0.0004	0.023 ± 0.0002	0.026 ± 0.0003
15%	15	ND	ND	ND	ND
	30	0.022 ± 0.0002	0.023 ± 0.0001	0.016 ± 0.0001	0.014 ± 0.0001
	60	0.040 ± 0.0003	0.032 ± 0.0002	0.022 ± 0.0001	0.014 ± 0.0001
	90	0.050 ± 0.0001	0.033 ± 0.0002	0.024 ± 0.0001	0.022 ± 0.0001
20%	15	0.012 ± 0.0001	0.014 ± 0.0001	0.014 ± 0.0002	0.012 ± 0.0001
	30	0.022 ± 0.0002	0.022 ± 0.0002	0.016 ± 0.0001	0.013 ± 0.0001
	60	0.034 ± 0.0003	0.025 ± 0.0002	0.021 ± 0.0002	0.021 ± 0.0001
	90	0.052 ± 0.0004	0.026 ± 0.0003	0.033 ± 0.0003	0.025 ± 0.0002

Data expressed as the mean ± standard deviation (SD) of the three independent biological replicates with *P* < 0.005; *ND* not detected

**Fig. 1:**
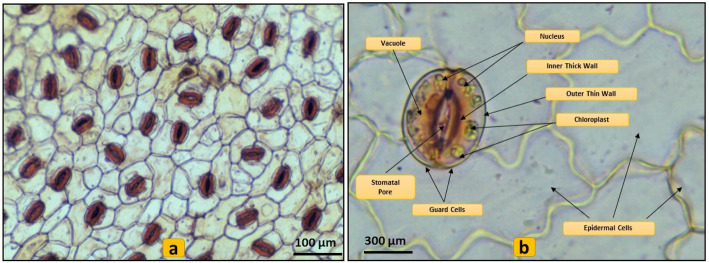
Microscopic study of epidermis. Stomatal density with anomocytic and anisocytic stomata (**a**). Structure of stomata (**b**)

**Fig. 2:**
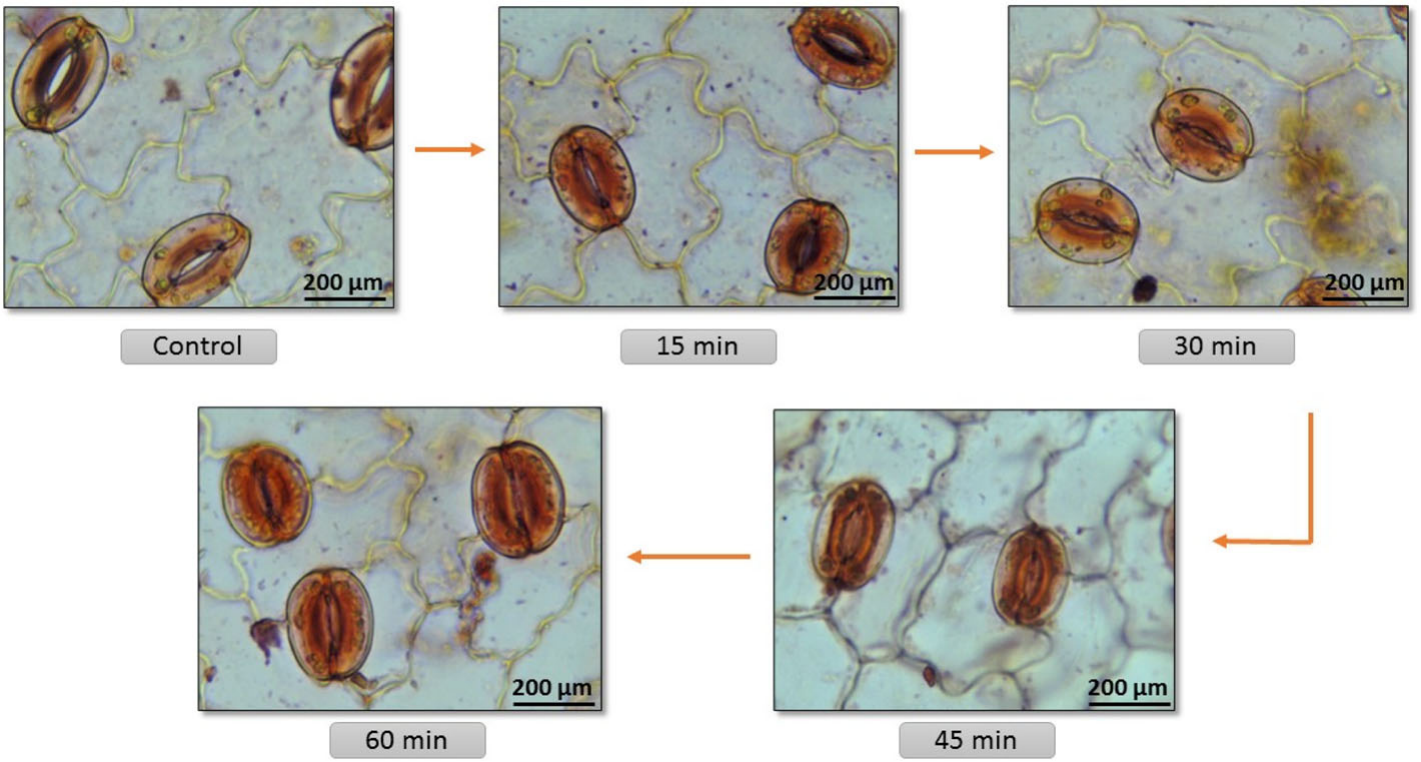
Stomata under 13% mannitol-mediated plasmolytic treatment. Open stomata (control). Closing gradually (15–30 min). Completely closed (45–60 min)

The microscopic examination of the leaf epidermis was carried out to understand the effect of plasmolysis on cells of leaf epidermal cells generally considered the site of organogenesis. The epidermis of *C. roseus* is found single-layered in all experimented leaves and the leaves contain thick-walled, irregular, and amoeboid epidermal cells bearing stomatal assembly with both anomocytic and anisocytic stomatal types (Fig. 1). Stomata are key organs responsible for gaseous exchange between the inside environment of leaves with outside air for photosynthesis along with water evaporation over transpiration [5]. The stomatal aperture inflection arises with the environmental-dependent variation in the osmotic potential of guard cells into water-fluxes and mechanical-forces that regulate the dimensions of stomatal pores [5]. Guard cells sense the osmotic pressure applied though predominant osmotically active species lie K^+^, malate, Cl^-^, sucrose, and other sugars, thus regulating the cell volume through changes in stomatal aperture [1, 7]. The mannitol used in the present study applied a successful positive osmotic stress leading to the gradual closing of stomata (Fig. 2). However, the guard cell turgor regulation against osmotic stress is even more complicated. While picking the leaves for the experimentation the stomata were opened conceivably due to the hindered gaseous exchange from the mouth-space of the culture vessel and to maintain the equilibrium with ambient in vitro environment, the stomata remained open [2]. Verma and Mathur [13] also proposed that plasmolysis of leaves with hypertonic mannitol solution before culturing over the shoot induction media to get the support of ex-osmosis to reduce toxic metabolites inside the cells. Later the same approach was used to generate transgenic *C. roseus* from pre-plasmolyzed leaves undergoing dedifferentiation and redifferentiation process for de novo shoot bud organogenesis [11, 14]. This approach can have wider implications in tissue culturing of all those medicinal plants that are known to accumulate cytotoxic molecules in their leaves or other explants. Alternately, this treatment can also influence organogenesis by the easy and rapid uptake of plant growth regulators by leaves culturing over the shoot bud induction media through an osmotic pull [13].

## Conclusion

The presented work has consolidated the observation concerning the positive influence of 13% (w/v) mannitol CPW solution-mediated leaf explant pre-plasmolysis to obtain direct bud organogenesis that could be very useful for genetic engineering of *C. roseus* at the whole-plant level.

## Data Availability

Not applicable.
